# Bio-Inspired Robots and Structures toward Fostering the Modernization of Agriculture

**DOI:** 10.3390/biomimetics7020069

**Published:** 2022-05-29

**Authors:** Maria Kondoyanni, Dimitrios Loukatos, Chrysanthos Maraveas, Christos Drosos, Konstantinos G. Arvanitis

**Affiliations:** 1 Department of Natural Resources Management and Agricultural Engineering, Agricultural University of Athens, 75 Iera Odos Str., Botanikos, 11855 Athens, Greece; mkondoyanni@aua.gr (M.K.); maraveas@aua.gr (C.M.); karvan@aua.gr (K.G.A.); 2 Department of Industrial Design and Production Engineering, University of West Attica, 250 Thivon & P. Ralli Str., 12241 Egaleo, Greece; drososx@uniwa.gr

**Keywords:** biomimetic, agriculture 4.0, intelligent materials, bio-inspired, IoT, machine learning, robotics

## Abstract

Biomimetics is the interdisciplinary cooperation of biology and technology that offers solutions to practical problems by analyzing biological systems and transferring their principles into applications. This review article focused on biomimetic innovations, including bio-inspired soft robots and swarm robots that could serve multiple functions, including the harvesting of fruits, pest control, and crop management. The research demonstrated commercially available biomimetic innovations, including robot bees by Arugga AI Farming and the Robotriks Traction Unit (RTU) precision farming equipment. Additionally, soft robotic systems have made it possible to mitigate the risk of surface bruises, rupture, the crushing destruction of plant tissue, and plastic deformation in the harvesting of fruits with a soft rind such as apples, cherries, pears, stone fruits, kiwifruit, mandarins, cucumbers, peaches, and pome. Even though the smart farming technologies, which were developed to mimic nature, could help prevent climate change and enhance the intensification of agriculture, there are concerns about long-term ecological impact, cost, and their inability to complement natural processes such as pollination. Despite the problems, the market for bio-inspired technologies with potential agricultural applications to modernize farming and solve the abovementioned challenges has increased exponentially. Future research and development should lead to low-cost FEA robotic grippers and FEA-tendon-driven grippers for crop harvesting. In brief, soft robots and swarm robotics have immense potential in agriculture.

## 1. Introduction

The term biomimetic in agriculture denotes the unique ability to interlock artificial and natural systems without altering the function and ecosystems of wild species by mimicking nature [1]; this is achieved through the adoption of an interdisciplinary approach in the development of machines, systems, and materials that are inspired by biological processes for scientific, engineering, and medical applications [2]. Even though research and development in biomimicry are new, the history of biomimicry can be traced back to Leonardo da Vinci’s flying machine, whose design was inspired by birds. Such inspirations led to the development of the first commercial aircraft, bionic car concepts, animal-shaped robots, and architectural techniques adapted to nature [2]. That the recent biomimetic synthesis of biomaterials for agriculture remains one of the most critical applications of biomimetics in agriculture is a case in point [3,4]. The biomaterials have a wide array of potential applications that range from hydrogels for water storage, carbon capture, biodegradable materials for greenhouses, antimicrobial packaging for fruits and vegetables [5,6,7], and sustainable agriculture. This review focuses on soft robotics and swarm robotics, which hold great promise in harvesting, plant management, and seeding [8,9,10,11]. The emphasis on soft robotics and robot-animal artifacts is expected to translate to a nuanced understanding of the benefits, technological challenges, and prospects. Such information would help guide decision-making among smallholder and large commercial farmers. Consumer demand would be a catalyst for industrial research and development.

Biomimetic-related innovations hold great promise in ecological system-design models [1] for the intelligent mitigation of soil degradation, the conservation of biological systems, and the conceptualization of smart farming technologies [12]. Smart farming technologies have been proven to reduce production costs and improve yields through the intelligent regulation of humidity, irrigation, frost, greenhouse microclimate, pesticide, and fertilizer applications [7,13,14]. Beyond production, biomimetics has practical applications in electronics (which mimic light-reflective butterfly wing structures), eco-friendly packaging, and the production of laboratory-grown meat [15]. The list is not exhaustive considering new applications for biomimetic-related applications are emerging with advances in research.

**Figure 1:**
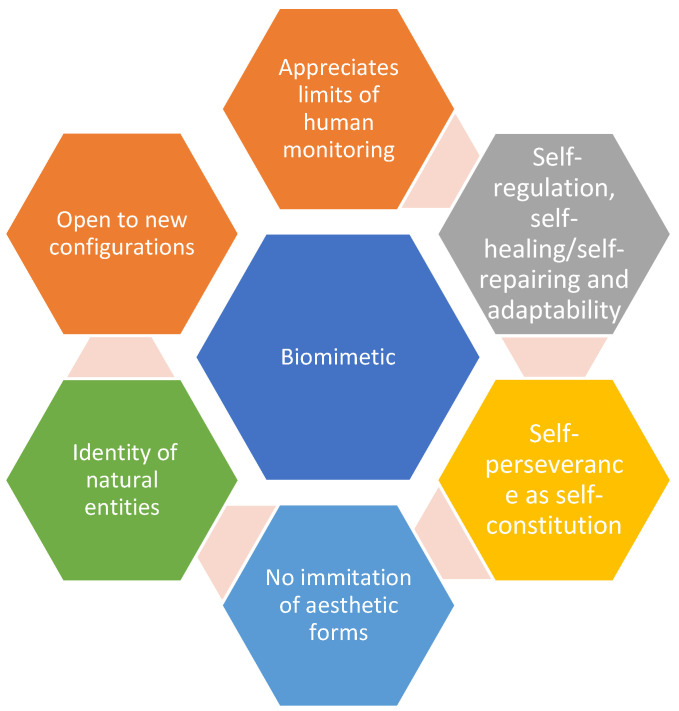
Unique attributes of biomimetics and contributions to agriculture and nature conservation [12].

Recent reports suggested that the biomimetic agriculture industry would grow exponentially and contribute to global economic development. On average, biomimetic-related innovations would contribute at least $1.5 trillion to the global domestic product [15]. However, the latter contribution would have a lesser adverse effect on local ecosystems because the biomimetic revolution learns and mimics nature; this is in contrast to the 19th-century industrial revolution, which exploited nature [1,12,15]. The unique contributions of biomimetics to agriculture are depicted in Figure 1. In light of the diverse potential contributions of biomimetics to agriculture, the review article focused on applications in commercial agriculture, particularly smart and intelligent IoT-mediated farming in Europe, the Middle East, Asia, and North America.

Biomimetic research and development by academia and industry would have a tangible impact on global food security. The focus on innovation to address global food security was justified by the scale of the challenge. According to the WHO, 12% of the global population is food insecure, and about one in three persons (2.37 billion) lacks access to adequate food [16]. Future projections indicate that 660 million persons will experience hunger by 2030 [17]. The projected food insecurity must be addressed because the global population will increase by 10% [18]. Higher demand for agricultural produce translates to a concomitant increase in price and inflation, which might translate to further food insecurity [18]. Drawing from the current state of food insecurity post-COVID-19 [19] and future projections, concerted measures are necessary to reverse the current trend of food insecurity.

Practical interventions include agriculture 4.0, IoT, and biomimetic agriculture. This review focuses on the potential of the latter, given the former was extensively reviewed by [20,21,22,23,24,25,26,27,28]. The link between global food security and biomimetic research is grounded on the progress made using biomimetic approaches to address water scarcity, using inexpensive and scalable Warka and water cone water from the atmosphere [28]. Additionally, biomimetic approaches have led to the development of biomimetic phosphate scavengers via quantum chemical studies of phosphate anions on small, intrinsically disordered peptides [29]. The latter study demonstrated that it was possible to improve global food security using phosphate scavengers while minimizing the negative effect of phosphate compounds on the environment. The biomimetic-related innovations highlighted by Othmani et al. [28] and Gruber et al. [29] were but a microcosm of the various innovations that could transform the future of agriculture.

The current research focuses on two interrelated biomimetic systems, namely swarm and soft robotics [30,31]. On the one hand, soft robotics was designed to achieve specific functions such as harvesting fragile fruits and vegetables, seeding, and crop management [30,32,33,34,35]. On the other hand, swarm intelligence is the collective behavior of undistributed, self-organizing physical or artificial systems. Applying swarm principles to robots is called swarm robotics, where robots aim to mimic natural swarms, such as ants and birds, to form a scalable, flexible, and robust system. Flocks may be considered as a type that can adapt to the environment’s changes and follows specific behavior [2,36,37], such as the fulfillment of objectives, aggregation or dispersing, communicating, and memorizing. Similarly, swarm robots exhibit autonomy, cooperation, and coordination, which are necessary for long-term applications. 

### 1.1. Global Agricultural Challenges and Need for Biomimetic Innovations 

Global agriculture has been constrained by many factors, including biophysical and socioeconomic issues, including climate change [38,39]. Climate change is a catalyst for desertification and diminished crop yields attributed to the depletion of vital nutrients in agricultural lands [38,39]. The United Nations Convention to Combat Desertification (UNCCD) estimated that 1.6–3.3 million ha of farmland would be lost annually due to urbanization. The pattern is anticipated to persist between 2000 and 2030 [40]. Other studies estimated that about 23 ha are lost per minute due to climate change-induced desertification [41]. Empirical evidence suggests that traditional interventions could not suffice because they had not helped to address the problem from the onset. Traditionally, commercial farms and smallholder farmers had sought to address global food insecurity through the intensification of farming. For example, the global production of essential foods such as cereals has surged by >200% compared to the 1960s [42]. From an economic perspective, there were practical challenges with this intervention. First, the intensification of agriculture did not translate to greater adoption of sustainable measures such as irrigation to save water; IPCC’s special report corroborated these claims. As of 2015, only 2% of global croplands were irrigated [42]. The limited effectiveness of the traditional measures reinforced the need for climate-smart agriculture and biomimetic innovations. 

Despite the compelling evidence supporting the transition, critics have argued that developing countries, including those in Sub-Sahara Africa, are less equipped for the transition to agriculture 4.0 [43]. Additionally, new research questions the development and rollout of robot bees that mimic natural insect/bee pollinators. In place of creating robotic free-flying bees and insects to facilitate pollination, it would be much more appropriate to restore natural ecosystems and “create environments that are friendly to bees and exploring the use of other species for pollination and bio-control” [10]. The observation represents one of many ethical dilemmas agricultural experts, scientists, and engineers face as they seek to optimize crop yields. The essence of equipping developing countries for the transition remains unclear, especially in the case of soft robotics. The argument is premised on the fact that soft robotics and seeding/planting equipment seek to address labor shortages inherent in Europe [35].

In contrast, developing nations in Africa have a critical mass of unemployed youth [44,45,46,47,48]. Drawing from the employment dynamics in the global north and south, labor shortage is not a sufficient incentive for the transition to soft robots and robot insects. However, efficiency in cultivation and harvesting is compelling.

The transition to agriculture 4.0 is necessary for developing nations because it offers unlimited potential by adopting robotic arms for harvesting and seeding, precision tractors, drones for the detection and location of fruits using UAV images [47], land tilling, fertilizer, and pesticide applications [49,50,51], and decision support systems based on machine learning techniques for tree health monitoring and fruit diseases classification [52,53,54]. The use of precision tractors serves multiple functions. First, it provides a practical solution to the labor shortage. Second, it reduces greenhouse gas emissions [49,50]. Third, the precision tractors are integral to intelligent and precision planting using tractors fitted with soft robots and guided by LIDAR (light/laser detection and ranging) or a global navigation satellite system [34,55]. The Robotics Traction Unit (RTU), which retails at £ 7000, is a case in point [56]. The RTU serves multiple functions, including harvesting and crop monitoring. Even small autonomous robotic systems can assist the harvesting process and be combined with recognition systems using deep learning for the fast and precise detection and collection of fruits [57]. The extent of preparedness is reviewed in the next section, emphasizing soft robotics and swarm robotics. 

### 1.2. Review Framework

**Figure 2:**
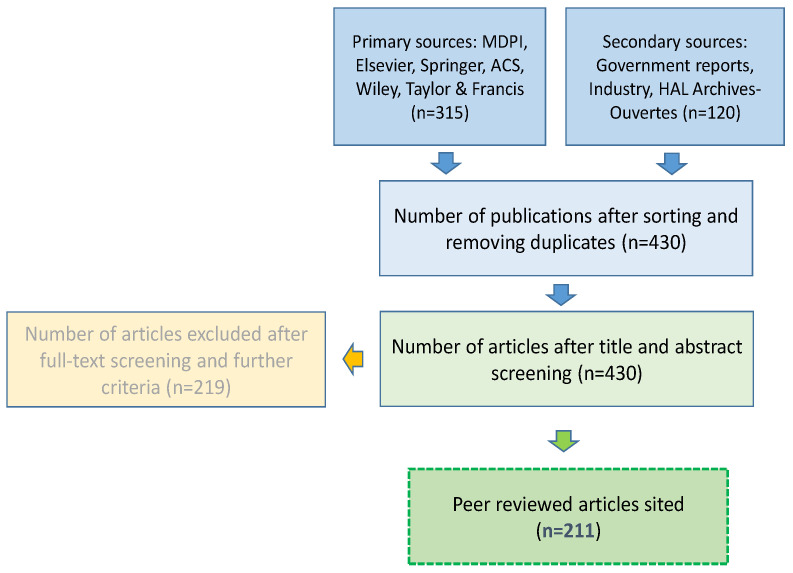
Diagram of the literature source selection process.

A rigorous review framework was adopted in the preparation of this review paper. The review process was aligned with the PRISMA guidelines for systematic reviews and meta-analyses (see Figure 2). The majority of data were sourced from published articles. More specifically, the peer-reviewed data were sourced from the following primary databases: MDPI, Elsevier, Springer, Wiley, and Taylor and Francis. Secondary sources were also taken into account, such as government reports published by the USDA and European Commission and industry stakeholders, including Boston Dynamics, Arugga AI, and Bird Gard Australia. The literature focuses on recent developments in biomimetics research and potential applications in agriculture. The articles were selected using keywords such as bio-inspired, biomimetics, biomimicry, agriculture, smart agriculture, solar panels, tropism, soft robotics, swarm robotic structures, and materials. The research was mainly focused on English-published papers to avoid translation delays. The publication period was between 2005 and 2022. The search window was justified considering it was necessary to understand how biomimicry has evolved over the years and how it can be applied in agriculture. The inclusion and exclusion criteria were characterized by a title and abstract screening followed by a full-text screening process, which focused on the relevance of the subject. Part of the grey literature was excluded because it did not satisfy the inclusion criteria. 

The reviewed articles were grouped into three categories: firstly, biomimetic innovations, including soft robots, swarm robotics, and other intelligent systems with broad applications in agriculture. The second and third classifications included biomimetic materials and resource management. The categorization followed a logical order of increasing complexity, starting from the natural resources necessary for the crops, continuing to the materials that are important for the construction of various farming uses, and finally mentioning more complex mechanisms that facilitate the processes. The bio-inspired technologies applied in resources management were divided into sub-categories, including solar energy harvest and water preservation.

## 2. Biomimetic Innovations and Climate-Smart Agriculture 

### 2.1. Intelligent Systems Connectivity and Cost

The introduction of IoT infrastructures greatly assists in making agricultural processes more efficient and accurate. These systems may utilize technologies from conventional cellular and Wi-Fi to long-range and low-rate radio transceivers [58]. The selection of components depends directly on the type of application and the information needed to be transferred and should be addressed meticulously. Even though soft robotics and swarm intelligence offer unique advantages compared to traditional systems, integration in farms is often impacted by cost and the lack of intelligent system connectivity [59,60]. Even though new research and development projects have attempted to address the problem, the issue has persisted. The problem is multidimensional, considering that intelligent systems are energy-intensive. A recent study estimated that the energy expenditure for large farms could be 65,891.5–151,220.6$ per year [61]. Such costs are unsustainable, considering farmers expect to achieve net savings of about $500/acre [62]. Based on the high costs of operating intelligent systems on farms, smallholder farmers are disadvantaged compared to large farms, which enjoy better economies of scale. 

The current research and development projects show that the technology and cost-related barriers to the adoption of biomimetic technologies could be addressed with time [2,12,28,29,63,64]. If existing R&D projects are successfully commercialized, swarm robotics could be employed on a broader scale to solve pressing challenges on farms. For example, efficiency, cost reduction, and the optimization of crop production have become a priority [13,65,66,67]. However, big data in decision-making remains a challenge [36]. The type of data that each farmer needs depends on the type of production. Water consumption, soil fertilizer levels, weather conditions, and crop growth are some useful data for a farmer. Combining all these data with artificial intelligence, farmers receive practical knowledge that gives them real-time updates. However, the use of many sensors is hampered by several factors related to connectivity. Commercial farms in developing countries and remote regions have inadequate access to the internet and infrastructure required to support intelligent machines; this explains why most innovations are concentrated in developed countries [66,67,68,69,70,71,72]. Wired sensors are less efficient than wireless sensors, which can automatically relay signals remotely. Refs. [73,74,75,76]. Mobile-based connectivity to the sensor network is less sustainable considering the high-power consumption and initial cost of the infrastructure. 

**Figure 3:**
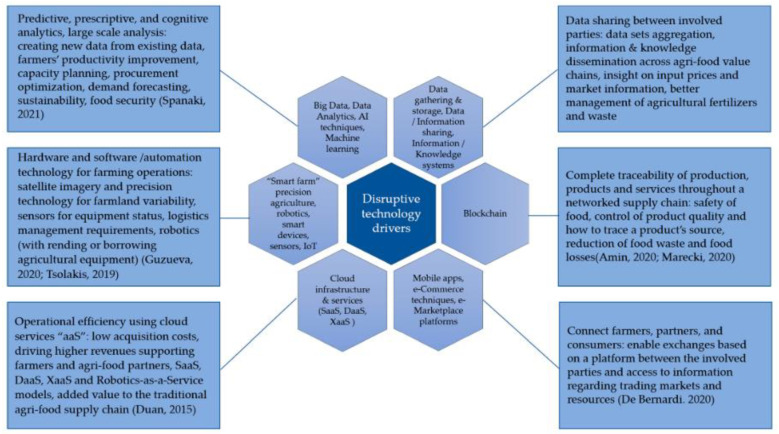
Relationship between disruptive technology drivers in the agricultural domain [81].

Biomimetic innovations in the agricultural sector cannot be considered outside the context of support technologies such as precision tractors [50,55], IoT [75,76,77,78,79], and LIDAR (light/laser detection and ranging) or global navigation satellite system [34,55]. The multidimensional view is grounded in the fact that soft robots and swarm intelligence cannot operate autonomously without the supporting infrastructure [80]. The worldviews advanced by Duckett et al. [80] were in agreement with Rial-Lovera [35], who claimed that real-time kinematics, GPS, actuators, specialized sensors, and interfaces were enablers for automation systems, particularly robots in agriculture. The integration of IoT and AI was evident in developing the first soft robot by researchers from Harvard University working in collaboration with Defense Advanced Research Projects Agency (DARPA) [33]. Drawing from past trends, future advances in soft robotics, swarm robotics, and biomimetic innovations, in general, would be predicted by developments in IoT and cloud computing, data storage, and big data, as illustrated in Figure 3 [81]. Beyond the disruptive technology, consumer attitudes and the ability to rapidly deploy the innovations would predict the rate at which soft robotic grippers replaced handpicking and mechanical harvesters.

The first subsection explores the cost benefits of soft robotic systems in agriculture. Based on the current body of knowledge, biomimetic innovations are a prerequisite for large-scale sustainable agriculture [34,35,49,55,80]; this has been demonstrated in advanced economies such as Australia, where commercial agriculture had led to optimal land-use—about 20% more land compared to traditional tractors. A study conducted by the University of New South Wales [55] observed that precision tractors replaced the traditional manual tractors, which were expensive and less effective considering they compacted the soil and created crop lines and large paddocks, which made 20% of the land unusable and contributed to the degradation of the land in the long-term; this problem can be significantly reduced using precision tractors.

### 2.2. Soft Robotics in Commercial Harvesting

**Figure 4:**
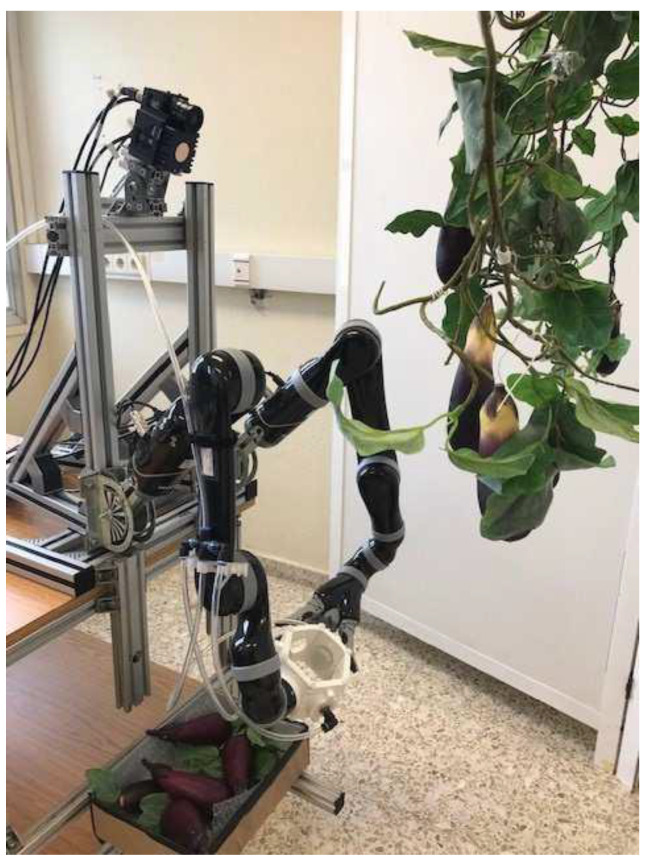
Soft gripper robotic arm designed in Spain for robotic agricultural harvesters [8].

Soft robotics is an emerging class of robots that easily adapt their form and shape to external obstacles and constraints and can easily deform (see Figure 4) [8]. In contrast to traditional rigid machines, soft robots have unique soft-touch capabilities, which are desirable for weeding, irrigation, fruit picking, and seeding [34,80,82]. The soft gripper robotic arm designed for a robotic agricultural harvester by researchers in Spain was comparable to a soft robotic arm developed by Chinese researchers. In the latter case, the soft robotic gripper mimicked the human hand—it relied on a simple control scheme with infinite degrees of freedom that enabled shape and size changes that matched the load in a wide range [83]. Despite the intelligent use of materials and systems, soft robotics is not immune to the mechanical damage of fruits and vegetables. The observation was in line with [84], who documented fruit damage at high grasp force and pressure. 

Navas et al. [8] and Yan et al. [83] concurred that soft robotic arms were better than traditional mechanical robots, vegetable and fruit grippers with rigid or underactuated grippers, which lacked precision control and often damaged the fruits. Mechanical damage to fruits and vegetables assumed different forms, such as surface bruises, rupture, crushing, plant tissue destruction, and plastic deformation. The probability of mechanical damage is higher in apples, cherries, pears, stone fruits, kiwifruit, mandarins, cucumbers, peaches, pome, and other fruits with a soft rind [84,85]. Integrating a bio-inspired robotic arm with high precision control mitigates the risk of fruit and vegetable damage during harvesting. 

Drawing from the evidence presented in Table 1, soft grippers are either fluidic elastomer actuators (FEAs) or FEA-tendon, primarily because these two technologies are best suited for agriculture because they rely on soft actuators to regulate the grip force. From an abstract perspective, the FEA is a suitable alternative considering it relies on affordable materials, which are easy and simple to manufacture. In addition, the grip strength of the FEA is appropriate for different types of fruits and vegetables, as shown in Table 1. Similarly, the FEA-tendon-driven soft gripper has a desirable payload to weight ratio of 7 kg and can lift a weight of 27 kg [86]. The constraints associated with FEA robotic grippers could be addressed by optimizing the gripper type, size, lifting ratio, scalability and controllability, response time, and surface conditions, among other parameters [86]. Considering the technology was nascent, optimized soft gripper systems are expensive and out of reach for smallholder farmers; this remains one of the key limitations of biomimetic innovations [87]. Other challenges include the prospecting of a suitable biomimicry pattern, relevance to the problem, information accessibility, and implementation on a broader scale.

Conservative estimates suggest that mechanical damage reduces the orange fruit quality; this is evident from the 5–11% lower ascorbic acid, titratable acidity, and soluble solids levels compared to undamaged fruits [88]. The post-harvest losses linked to mechanical damage have serious economic implications. For example, in the UK and the US, the cost of harvesting and post-harvest losses are between 18 and 24 billion pounds [89]. The volume of post-harvest losses could reduce with time, considering that the UK is making commendable investments in soft robotics and autonomous systems [80], making it a pioneer in intelligent technologies to reduce the demand for human labor and enhance production efficiency. For example, deploying the RTU eliminated the need for expensive human labor while minimizing the risk of errors [56]. On the downside, investing in soft robotics in isolation might not be a long-term solution because of other contributing factors. Reducing the risk of fruit and vegetable damage must consider that damage during harvesting remains a challenge considering there are other contributing factors, including the storage temperature, microbial infections, high temperatures, and relative humidity [85,88]. In light of the latter challenges, one can argue that the intelligent control of the grip force and pressure in robotic grippers alone could not eliminate the risk of mechanical damage. The storage and transportation conditions must satisfy the requirements.

Fruit and vegetable picking grippers are an important technology to achieve rapid and labor-saving harvest. However, most of the existing fruit and vegetable picking grippers still use traditional rigid or underactuated grippers, which often cause fruit and vegetable damage by the heavy mass and lack of high-precision control, and have poor compliance in the operation process [55,82,83,84,85]. In recent years, inspired by soft creatures’ tentacles, soft robotic grippers have appeared and been used in robotics due to the emergence of soft robots. The soft robotic gripper is made of flexible material integrated into a smart autonomous system that regulates the grip force and pressure. The risk of mechanical damage using mechanical harvesters versus soft robots is illustrated in Table 2. Following the comparative analysis of mechanical harvesters, handpicking and robotic systems, there was a lower risk of fruit damage in the latter. 

Preliminary research conducted by Chowdhary et al. (2019) showed that soft robotics (small bots with soft arms) could perform a wide range of complex tasks on farms. The commercial rollout of the soft robots is feasible, considering the robots are affordable and effective compared to the traditional hard robots [8]. Despite the huge potential of soft robotics in agriculture, the level of adoption remains low, and most projects are in the pilot phase, such as the swarm robotics for agricultural applications (SAGA) project [90] and Soft Robotics LLC’s SoftAI™ powered robotic solution, which combines AI, 3D vision, and IP69K-rated soft grasping technology [91]. According to Soft Robotics LLC, the robots have comparable hand-eye coordination to humans [91]. On the downside, there is minimal data from commercial companies that have adopted the technology.

The inadequate commercialization of the technology could be linked to the newness of the technology. As of 2021, Soft Robotics LLC sought Series B funding from investors to enhance operations. From a theoretical point of view, the limited uptake of soft robots could mirror earlier reservations about the adoption of precision agriculture across Europe [49]. During the 1990s, the adoption of ICTs, particularly precision agriculture, was confined to Canada and the US, primarily because there were large smallholder farms with properly organized extension services by university research teams and government extension officers [49]. In addition, the investments in precision agriculture at the time were mediated by consumers’ willingness to explore new technologies, higher incomes, access to financial resources and investments, and subsidies. On the downside, the positive growth in precision agriculture decreased in the early 2000s due to many challenges. First, certain technology systems were incompatible across brands, and consumers expressed reservations about the ease of use and maintenance, fuel use, and projected productivity. The demand for precision agriculture has resurged with the demand for sustainable agriculture and the judicious use of fertilizers, pesticides, and water. 

The problems experienced in the transition toward precision agriculture are comparable to the current scenario. Leading soft robotic system manufacturers have experienced significant challenges transitioning from laboratory to commercial adoption [30]. The closure of Empire Robotics—a market leader in soft robotics—is a case in point. Despite spending significant resources on R&D and releasing a revolutionary product, VERSABALL^®^, the company was unable to achieve a sustainable business model [30]. The case is but a microcosm of the challenges faced by companies in soft robotic systems. The challenge could be attributed to unique market dynamics, such as a new value chain mediated by data analytics, robotic machines, aerial imagery, and disruptive technology-mediated agrifood-technology e-business models [81,92]. Business theories developed to delineate the antecedents and barriers to new technology adoption offer a nuanced understanding of why certain consumers (commercial farms) were hesitant. One of the plausible explanations is that the new technology did not provide sufficient relative advantages or did not elicit pleasure and arousal based on the perceived ease of use and usefulness. These variables predict positive or negative attitudes toward new technologies and adoption intentions [93]. Based on the theory of technology adoption, there is a need for customized soft robotics and swarm robotic solutions that address the unique needs of commercial farms. The view is further supported by the fact that the harvesting of cherries, pears, stone fruits, kiwifruit, mandarins, cucumbers, peaches, pome fruits, and tomatoes requires different grip force and pressure and biomimetic designs based on the shape and size of the produce [84,85]. The challenge of developing efficient pickers for fruit collection, demands systems that, apart from having the suitable electromechanical characteristics, should be equipped with fast and accurate fruit identification algorithms exploiting machine vision and other techniques [47], such as the one destined for peaches [94] or the one for banana recognition and cutting [57]. Machine vision imitates the ability of most animals to see, while stereo vision is inspired by the calculation of depth information from the views of different points in space, which was thought to be confined only to primates and mammals but has also been demonstrated in other animals, such as birds and amphibians [95]. These artificial vision mechanisms can be used to enhance the actual systems serving agricultural tasks [96], including maturity detection for ripe fruit harvesting [57,97] or for vegetables [98] and similar classification actions [99,100]. Pest and disease detection is also a process typically based on visual techniques [52,101,102] and has a direct impact on the quality and quantity of the fruits being collected. Furthermore, automatic navigation and obstacle detection based on visual data [103,104] are important functions for supporting the system carrying the gripper effectors or performing the fruit transportation or plant treatment tasks. For these reasons, apart from the soft robotic mechanisms for harvesting, the current state of the adoption of swarm robotics is reviewed in the next sections. 

### 2.3. Swarm Robotics and Robot Bees

**Figure 5:**
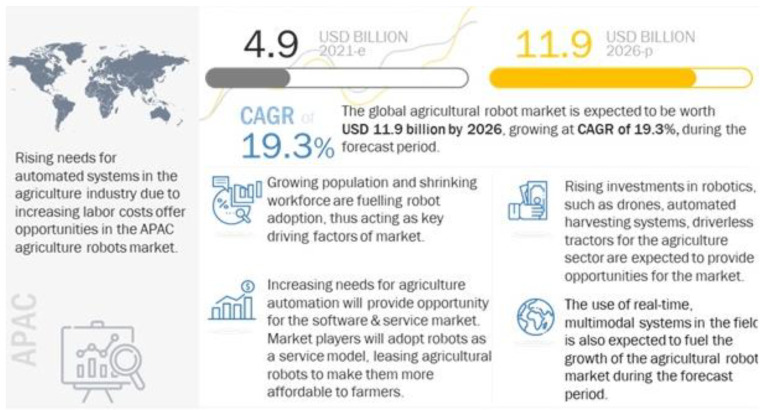
The current state of the global swarm robotics market [109].

The term “swarm robotics” refers to a combination of different micro-robotic systems, simple and homogeneous or heterogeneous agents, coordinated in a decentralized and distributed manner [105]. The development of swarm robot bees is a case in point [105,106,107]. Swarm robotics is unique because they lack a centralized control and act according to simple and local behavior, and this behavior is capable of solving complex tasks. The fundamental characteristics of swarm robotics include autonomy and the ability to cooperate to solve different tasks. The autonomy of robotic systems is important because it predicts the commercial application of swarm robotics. Recent industry data estimated that the cumulative annual growth rate would be about 9.9% and market capitalization would exceed $81 billion [108]. The projections made by Bluewave Consulting [108] were in line with other market reports, which forecasted a CAGR of 19%. However, the market value in the latter case was estimated at $11 billion [109]. The current state of the swarm robotics global market is illustrated in Figure 5. According to Figure 5, market growth was reinforced by investments in robotic systems, the consumer adoption of real-time multimodal systems, and agricultural automation. 

The growing adoption of robots in agriculture would inadvertently lead to complex robot–animal interactions and animal–robot mixed societies. Recent studies have focused on human–computer interaction (HCI) to mitigate the adverse effects of human–robot interactions [110]. The preliminary evidence from published scholarly studies demonstrated it was practical to build micro-robots that could fit into the cockroach ecosystem [110]; this was a gateway to the higher deployment of micro-robots in pest control and soil health monitoring. Such findings could pave the way for the greater adoption of micro-robots that live harmoniously with bees and other insects that influence crop pollination [111]. The development of precision robots for agriculture is anticipated to increase with the development of micro-robots, which can serve unique functions. Researchers at the University of Exeter had successfully developed bio-inspired bio-hybrid micro-robots that combined biological and synthetic components, which enabled them to perform tailored biochemical operations with precision at the nanoscale [112]. Such microbe-mimicking robots could have unique functions in agriculture, such as monitoring plant health and eliminating pests and disease/pest control.

From an R&D perspective, the optimal potential of swarm robotics has not been fully exploited, considering there is growing interest in mimicking nature by learning from bacterial colonies, which continuously benefit from multicellular cooperation, facilitating cell division and the deployment of effective defense mechanisms [113]. Moreover, ants, bee colonies, and bird crowds offer useful insights into creating bio-inspired swarm robotic systems [113]. The limited exploitation of the full market potential might partly explain why industry growth is concentrated in the west while developing countries lag behind in the assimilation of swarm intelligence [108,109]. The delayed investment into swarm robotics can be regarded as a non-issue since developing countries in Africa and South Asia have vast colonies of honey bees, which are less endangered than those in Europe. A report by the IUCN noted that 9% of European bees were threatened with extinction [114]. The threat to natural bees underscored the urgency to invest in robot bees. The region-specific variations in the development and adoption of swarm robotics and other innovations in agriculture could be resolved with advances in new technologies.

**Figure 6:**
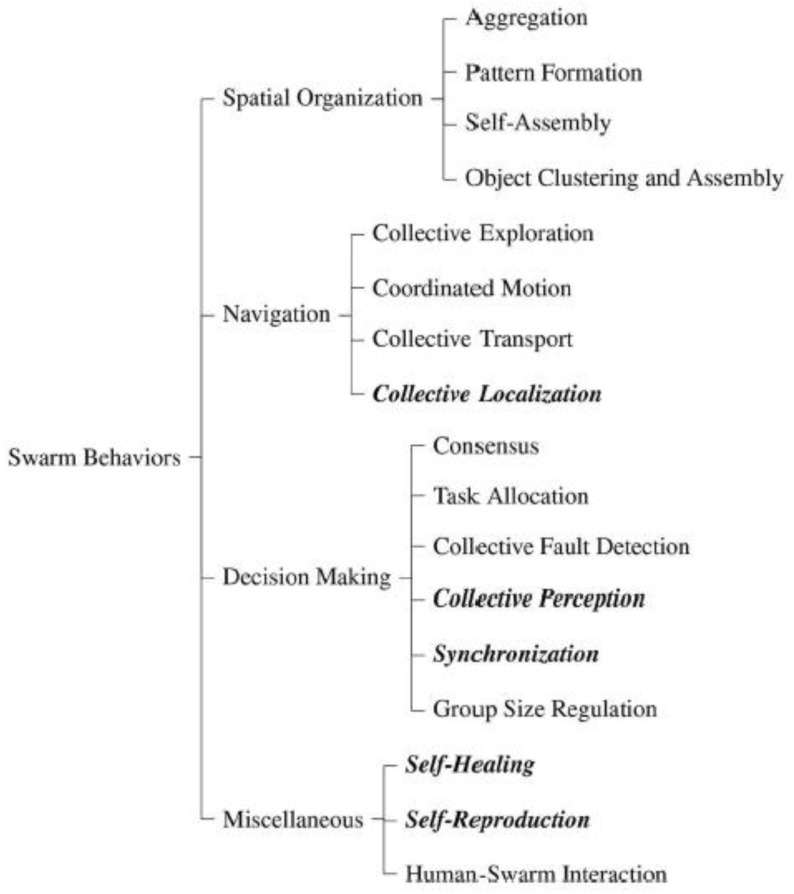
The function of different robotic systems [116].

The main advantages of swarms are adaptability, robustness, and scalability. These robots can operate without any central entity to control them, and the communication between the robots can either be direct (robot-to-robot) or indirect (robot-to-environment) [109,115]. Swarm robotics has a variety of applications, from simple household tasks to military missions. In a swarm, multiple robots, which can be characterized as homogeneous or heterogeneous, are interconnected to form a swarm of robots, which serve a variety of applications in the field of smart agriculture, such as sowing/seeding, the diagnosis of soil and plants, and irrigation systems [9,90]. Since individual robots have processing, communication, and sensing capabilities locally on board, they can interact with each other and react to the environment autonomously. The unique capabilities make it possible for swarm robots to replicate the reactions and functions of various swarms in nature (birds, fish, and insects) to carry out a specific goal. The perceived advantages and disadvantages of swarm robotic systems are largely contingent on the desired functions, including navigation, miscellaneous (self-healing, self-reproduction, and human swarm interaction), and spatial organization (see Figure 6) [116]. Considering that each sub-class of swarm robots offers unique benefits in agriculture, the adoption should be guided by local needs, technology adaptability, cost, and sustainability.

A comparison of different R&D projects and commercial applications of robotic systems in agriculture demonstrated that the swarm behaviors and availability predicted use. For example, swarm behaviors such as human–swarm interactions, partial self-healing, collective perception, task allocation, coordinated motion, collective exploration, and aggregation were associated with swarm robotics for terrestrial applications. On the contrary, swarm robotic self-assembly was best suited for aerial applications [116]. The observations made by Schranz et al. [116] were in agreement with other scholarly studies, which investigated swarm behaviors and intelligence [36,37]. Table 3 summarizes the link between different swarm behaviors, application and adoption, and environment. However, a fundamental constraint relates to the high costs of swarm intelligence systems and the lack of consensus about the ecological impact. As noted in the preceding sections, critics expressed reservations about the ecological impact of swarm robots [10]. However, the SAGA project suggests otherwise [90]. Despite the lack of consensus among scholars and industry stakeholders, swarm robotics have been employed in the field to facilitate automation in agriculture and another sector [36,37,113,115,116]. Based on the current market trends, it could be argued that ecological concerns were not a critical impediment to the adoption of swarm robotic systems.

Current research and development projects coupled with the scheduled projects might potentially enhance and reduce the costs associated with swarm agriculture [36,37,113,115,116]. The utility of biomimetic innovations would be augmented by advances in UAVs for agricultural applications. UAVs have been extensively used to provide aerial monitoring capabilities on farms, crop monitoring, and the spraying of fertilizers [43,109,117]. Despite the inadequate distribution of technological innovations, the progress would have a tangible and positive impact on farming in the agriculture 4.0 phase, given the development of light UAVs, which are quicker, affordable, and capable of providing real-time information of all sections in a farm. On the downside, developing countries with inadequate access to resources and intelligent systems would lag behind compared to developed countries.

#### 2.3.1. Case Studies of Commercial Adoption

The SAGA project was one of the most successful swarm robotics adoption case studies [90,116]. Beyond SAGA, researchers have developed an abstract model for weed monitoring [36]. The system aims to take over the monitoring task, generate task maps for future autonomous weeding robots, tell them which areas to work and plan their paths, and provide monitoring and mapping systems by swarms of UAVs. The system functions by exploiting a simple random walk strategy that constitutes a baseline. The baseline monitoring strategy against a disparate weed distribution is efficient, behaves well with low rates of weed detection, and presents good scalability with the group size [36]. The observations made by Albani et al. [36] were in agreement with Carbone et al., who noted that swarm robots were ideal for crop monitoring in precision agriculture [118]. Based on the preliminary positive evidence, UAVs can help farmers take aerial images of the crops, which can be processed to extract information about the state of the crops; this was demonstrated in the Mobile Agricultural Robot Swarms (MARS) project for farming operations [9]. MARS’s path planning, optimization, and the supervision of the robotic swarms are coordinated by a centralized entity called OptiVisor, which can supervise the seeding process, avoid collision between the robots, and react to robot failures.

The SAGA and MARS projects affirm that swarm-forming robots can improve crops and reduce environmental impact. Low-input robots offer potential such as performing tasks in the field that originally required the precise work attributed to human presence [36,37,113,115,116]. However, as they will potentially become increasingly integrated into human society, new regulations should be developed to govern the operations of swarm robotic systems. At present, there are no regulations, given the industry is still nascent [111,112,119,120]. Additionally, the development of advanced robotic systems is not confined to the agricultural sector in isolation; there are micro-robots for medical, industrial, and agricultural applications. Considering each sector has unique requirements, industry-specific regulations are needed.

**Figure 7:**
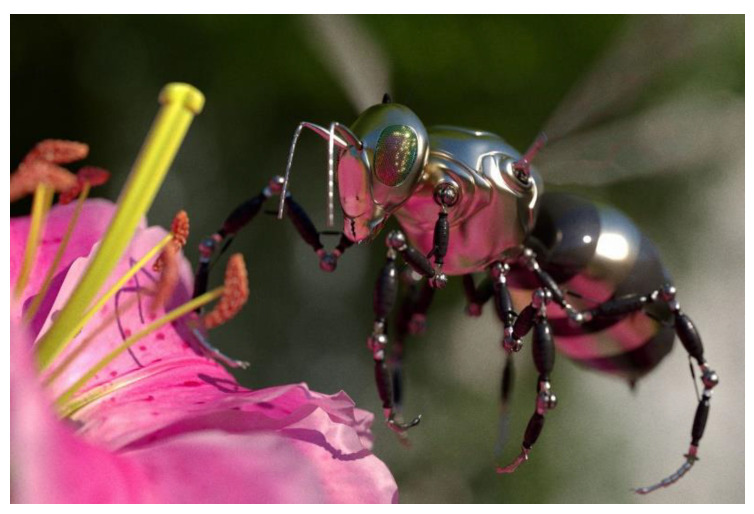
Robot bees developed by Arugga AI Farming Israel for cross-pollination.

In 2021, researchers successfully developed micro-robots that successfully mimicked bees and spiders. Of particular importance was the development of robot bees by Arugga AI Farming (see Figure 7), which exploit deep learning to facilitate cross-pollination in plants [120]. Even though the technology has not been widely adopted in the agricultural sector, preliminary data indicates that the robots could be more effective than natural bees, hand pollination, and flowers to attract bees [119]. The case for robotic bees advanced by Arugga AI Farming [119] was corroborated by Boffey [121], who suggested that the robot bees could complement the activities of natural bees, which are threatened by anthropogenic activities. There are valid economic grounds for the technology. For example, bee pollination contributed about $29 billion to the agricultural industry [122]. However, the economic contribution of honey bees is threatened by the pesticide poisoning of colonies.

Despite the strong support for robot bees in agriculture, critics raised concerns about the suitability of robot bees. One school of thought claims that robot bees could not replace natural biodiversity [123]. This worldview is grounded in the possibility that the robot bees could become an invasive species. Additionally, it is not practical for robot bees to entirely replace natural bees, considering mass production would have a negative ecological impact, and they cannot satisfy the cultural and intrinsic worth [123]. For example, bee-mediated pollination is essential to the proper function of the natural environment. 

Potts et al.’s [123] criticism was further reinforced by evidence collected by the University of Sussex Gould’s laboratory researchers, who claimed that it was highly improbable for humans to develop robot bees that are as effective as natural bees because bees had been “pollinating flowers for more than 120 million years; they have evolved to become very good at it. It is remarkable hubris to think we can improve on that” [124]. Economic considerations also justified the case against robot bees. 

As of 2020, there were about 3.2 trillion bees, which are self-sufficient and independent. If humans opted to replace the bees with robot bees, it would cost 32 billion assuming that each bee costs one penny (which is highly unlikely). The sustainability of the robot bees would be problematic, given robots cannot reproduce and often malfunction. In brief, the costs associated with replacing natural bees with robot bees are unsustainable. The negative economic and ecological arguments could be addressed with advances in technology, given companies are exploring the use of ecologically benign materials that are light, cheap, and biodegradable.

#### 2.3.2. Swarm Robotic Systems for Intelligent Pesticide Application 

Beyond the robot bees, researchers have developed swarm robotic systems for pest control [107]. The case for intelligent pest control is further augmented by the long-term and adverse cost implications associated with pesticide use. On average, 360 million kilograms of pesticides are sprayed on crops each year [107]. Despite the drawbacks mentioned above, the continued use of pesticides is encouraged due to the high returns on investment and lack of practical alternatives. Still, there are critical drawbacks linked to traditional pesticide application, given the method is imprecise, expensive, and inaccurate. It is not suitable for small farms and cannot be applied on farms with a mountainous terrain [125]. Moreover, large UAVs in pesticide application exhibit adjuvant-specific drift rates of 30–74% [126]. The higher the drift, the lower the precision and the higher the volume of pesticide wasted. 

There is a growing interest in delivering spraying robots with additional features, like thermal imagery capabilities for plant inspection and early disease detection [127], thus improving their autonomous characteristics and usability. Recently, researchers discovered that it was possible to develop swarm robots for pesticide application [107,128,129]. In particular, Skyx swarm technology has developed software for “autonomous and modular spraying robots” [130], while Greenfield Robotics has developed the hardware components for the wide-scale adoption of swarm robotic systems for pesticide applications [131]. The sustainability of traditional fertilizer application methods is questionable, considering the deleterious effect on the environment and human health. For example, pesticides have endangered nearly 10% of European bees, placed on the IUCN red list [114]. A primary concern is the inadequate data concerning applying the technology in real-life situations (smallholder and large farms). The observations made in the literature were drawn from pilot studies and experiments. In the absence of conclusive data comparable to robot bees, the use of swarm robotic systems in pesticide application would remain limited. The view was further reinforced by the fact that mobility and adaptability are impacted by unexpected changes in atmospheric conditions and obstacles, common in farms situated in the mountainous regions of South Korea, Japan, and China [126]. Swarm robotics might exhibit an undesirable phase transition between two macroscopic states [132]. The weather-dependent technical constraints should be addressed in future research and development projects.

#### 2.3.3. Robot-Animal Artifacts 

The advances in swarm and soft robotics have led to the adoption of different robot-animal systems, such as self-assembly systems [113]. The artifact mimics the different natural processes or structures of microorganisms and exhibits similar behaviors. Although such constructions seem impressive, they raise many questions about ethics, viability, and ecological impact [10]. Pollination is an essential part of plant reproduction and is mainly performed by bees [10]. Pollinating animals travel from plant to plant, carrying pollen on their bodies and transferring the genetic material, which is a vital interaction for the reproduction of most flowers. Robo-bees are small, autonomous aerial vehicles, weighing up to 10 g and sized just a few centimeters, which can replace the pollination activities of insects. These UAVs resemble bees and are designed to perform similar functions. However, the use of robo-bees raises ethical, economic, and ecological issues as noted in the preceding sections [2,12,29,64,87]. Nonetheless, there is strong support for bio-inspired innovations in agriculture to boost efficiency and optimize crop yields.

Fruits and vegetables depend on animal pollinators such as bees [10,106,120,133]. The replacement of natural bees with robot bees might disrupt natural ecosystems [86,113,134]. The production of robo-bees involves the assembly of electronic components made of metals, plastics, silicon, liquid crystal elastomers, stretchy polymer networks, and lithium, among other elements [135,136]. The processing of these elements from natural ores and the production of lithium polymer batteries to power the soft robots and swarm robotic systems releases toxic substances into the environment. As of 2019, the production (including mining, refining, and cell assembly) and operation of Li-Po batteries released about 70–110 kg CO2e/kWh [137]. Beyond production, there are other risks associated with the non-renewable and toxic materials in robots; for example, the swarm robots can fail or malfunction during operation [37,105,113,116,118]; this might result in the contamination of crops with toxic metals and elements. Even though there are legitimate concerns about ecological impact, the potential impact on production efficiency catalyzed widespread adoption. 

Market growth should not negate the adoption of standards to guide the responsible use of soft robotics and swarm robotics; there is a profound risk of the contamination of fruits and vegetables if the swarm robotics fall and release toxins. The risk remains significant regardless of whether the robot bees fail during operation, are damaged by weather, or fail to return to base [10]. Beyond weather related-issues, there are concerns about the life-cycle analysis of the innovations, considering most are made using non-renewable materials—hence the call to balance the use of materials in swarm robotics [138]. On the contrary, the swarm and soft robotics proponents argue that natural birds and pests equally damage fruits and vegetables. One study estimated that birds were directly responsible for $189 million in damages to crops and vegetables [139]. The estimates provided by Anderson et al. [139] were comparable to USDA’s Specialty Crop Research Initiative report, which established that wine grapes, blueberries, cherries, tart, and sweet cherries were highly susceptible to bird damage, which accounted for 13–67% of the losses (see Table 4) [140]. Adopting advanced crop management methods has not prevented bird damage to crops. Since natural and eco-friendly birds had a negative impact on high-value commercial crops, the potential risk of swarm robot system failure should not be a barrier to the widespread use of advanced technologies on farms; this is because bio-inspired management strategies have proven effective.

The USDA recommends the following bird damage prevention strategies: the modification of agricultural practices, better habitat management, decoy crops, the use of frightening physical devices, and chemical repellents [140,141]. Each of these strategies has been proven effective in preventing bird damage. On the downside, farmers are less incentivized to engage in bird management strategies unless the damage is severe due to the cost implications. Considering the cost implications associated with the management of birds, bio-inspired solutions have been proven to be practical alternatives.

Muller et al. reported the successful development of a bio-inspired autonomous aircraft for bird management [142]. However, the commercial rollout was constrained by the trade-off between the use of lightweight materials, structural strength, moment of inertia, and center of gravity [132,136,142]. Further improvements in design were necessary before commercial deployment. In the meantime, researchers have reported positive outcomes with the deployment of Bird Gard in Australia [143]. The Bird Gard system features a bio-inspired sonic and ultrasonic microchip technology, which is non-harmful, non-toxic, environmentally friendly, and humane. The system is ranked among the leading bird deterrence and pest control systems [143].

In contrast to the traditional methods employed to prevent bird damage, the system is affordable. Additionally, the miniaturized electronics and the high-energy-density lithium batteries make using small UAVs for biomimetic bird damage control in agriculture possible. The biomimetic systems mimic flapping wings, natural sounds, and behaviors, apart from exploiting ultrasonics. Recently, researchers have proposed developing a system that integrates all four capabilities in a natural predator-like design. In parallel, scientists concentrate their efforts in order to provide optimized path planning solutions for the UAVs destined for bird monitoring and repelling purposes [144]. 

**Figure 8:**
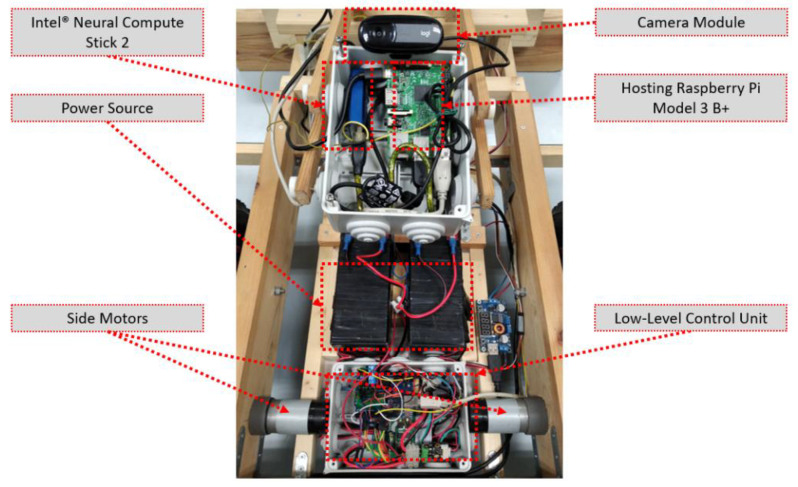
Details of the core engine of the robot presented in [145].

**Figure 9:**
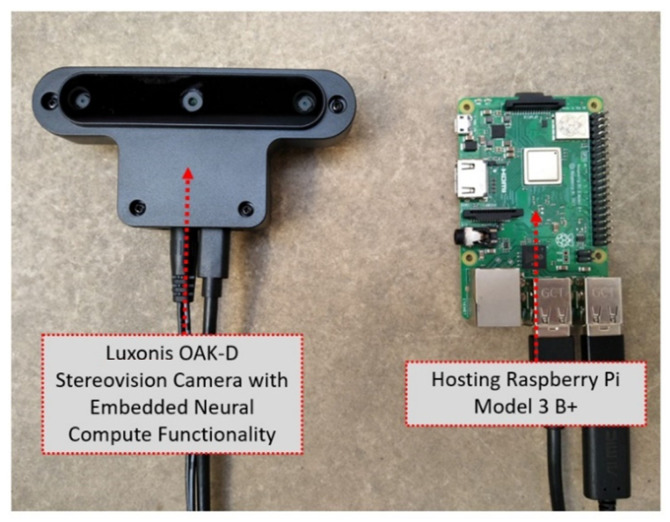
A Luxonis OAK-D stereovision camera that incorporates neural compute functionality, as an implementation variant enhancing the robot presented in [145].

Beyond the bio-inspired bird management systems, various farm activities can provide the basis for experimentation with biomimetic systems in agriculture, such as human- and path-following robotic mules or shepherd robots [145,146,147,148,149]. These systems may include monocular, stereo, or thermal vision combined with various assistive components, such as voice recognizers or neural network operation accelerators. Figure 8 shows details of the core engine of the robot presented in [145], where the machine vision process on a raspberry pi unit is assisted by an Intel^®^ Neural Compute Stick 2 accelerator chip, while details of the electromechanical actuators and controllers are also depicted. Figure 9 shows a Luxonis OAK-D stereovision camera that incorporates the same chip with the Intel^®^ Neural Compute Stick 2 for faster processing, thus leaving enough processing power on the hosting raspberry unit available for serving complementary tasks. 

In a more industrialized layout, Rocos and Boston Dynamics developed a remote-control system for robots that can herd sheep, assist in harvesting, inspect crop yields, and create real-time terrain maps. Apart from the advanced robots developed by Boston Dynamics, Australia’s SwagBot affords similar capabilities. The robot features a camera and a drone, which can be launched and work together in a two-bot team [148,149]. The shepherd robot can herd and interact with animals, traverse obstacles, drive over rough terrain and shallow water, and climb hills.

In contrast to the Boston Dynamics robot, the SwagBot was released in 2016, and further improvements have been made with the release of new and advanced models [148,149]. On the downside, there is no consensus on whether wheeled or legged robots should be deployed on farms. The legged robots were better at avoiding obstacles but performed tasks at lower speeds. In contrast, the wheeled robotic systems were fast but unable to overcome obstacles in their paths [150]. Field experiments conducted on the above robotic artifacts and similar ones show that they need improvement in terms of energy efficiency [150,151]. Beyond the energy requirements of the robotic systems, the broad adoption of robots in the agriculture sector raises economic, ethical, and environmental issues, as noted earlier. 

Future research and development should include establishing the policy choices necessary to meet the ethical challenges and maximize the benefits of the utilization of robots in agriculture. Other concerns raised by stakeholders include the absence of an effective robotic herding algorithm that can function optimally with a large herd of animals and nature [151]. Farm animals are not familiar with robotic systems, and it would take time to build robot-to-animal interactions and a suitable robotic herding platform [151]. The concerns raised by Li et al. [151] were in line with Fue et al.’s [150] research, which affirmed that bio-inspired robotic systems could not outperform humans in the short term. In brief, bio-inspired robotic systems can complement human labor.

## 3. Biomimetic Materials, Structures, and Resource Management

The choice of suitable materials for agricultural structures predicts productivity, operational costs, durability, and the sustainability of operations; this is because materials predict heat losses, insulation, and the absorption of UV radiation, humidity, and temperature [61,152,153,154]. Significant attention has been directed toward selecting suitable materials for livestock structures, farming equipment, silos for seeds and crops, as well as for greenhouses and their frames and covers [13,128,155,156]. Traditional farm buildings were designed as industrial buildings, using stone, brick, steel, or even timber; however, new structures are fitted with sensors and IoT systems for better regulation of the microclimate [24,61,153,154,157]. However, the risk of corrosion and material failure catalyzed the demand for bio-inspired materials that can withstand severe environmental conditions [158]. A wide array of biomimetic materials have been developed that can adapt and respond to their environment due to their unique deformable, elastic, and rheological properties [158]. The progress made in agricultural materials engineering could facilitate the creation of a wide range of smart materials and structures that can self-sense and self-repair without external intervention, such as microcapsules, calcite-precipitating bacteria, and shape memory polymers for fluidic elastomer actuators (FEAs) [86,159,160]. Preliminary data drawn from trials undertaken using self-healing systems/materials are promising [2,161]. The advantages include lower costs, better reinforcement, a lower risk of mechanical damage, and durability [12,116,158]. Specific examples of bio-inspired materials are reviewed under Section 3.1.

### 3.1. Biomimetic Materials

The growth of bio-inspired innovations is contingent on suitable materials to limit the adverse ecological impact and carbon footprint [159,162]. Progressive research and development have led to novel, lightweight and durable materials with bio-inspired relaxation and dynamic, multi-functionality, and hierarchical properties. The ideal materials include polymers and composites, and surfactants [87,159,162], biological materials, gels, and elastomers with easily deformable, elastic, and rheological properties [158]. The impact resistance of biological materials is predicted by the presence or absence of hierarchical structures, composites, porous structures, and interfaces. Other requirements include soft properties, especially for soft robotic grippers used to harvest fruits and vegetables. The soft properties are comparable to soft matter found in nature, such as collagen in human bones [158]. The soft body properties mitigate mechanical damage. However, the unique properties have substantial cost implications. The bio-inspired soft materials are expensive because they embody artificial intelligence in contrast to rigid-bodied robots [87,159,162]. For example, soft robots are integrated with robot operating systems (ROS) [160], deep learning systems, and RGB-D cameras with GPU computers [163]. Recent developments have demonstrated that the materials can be customized to achieve the desired function in agriculture. 

Conductive polymer materials are suitable for robot bees because they are light and durable. In addition, the polymers have superconductive properties and are elastic [159,164,165]. Other materials that have been developed for robots include shape-memory alloys (SMA) and magnetic shape memory (MSM) alloys or ferromagnetic shape memory alloys (FSMA) for micro-positioning applications [166]. Despite the progress made in developing advanced materials for robots, the cost of the materials remains a challenge, and this part helps explain the high cost of robotic materials. 

Another application is bio-inspired by biological compositions. An attempt to improve the mechanical properties of low-carbon steel with biomimetic units was made using a laser re-melting process and augmented by computer-aided design [167]. The unique benefits associated with the process were ascribed to the combined effects of the microstructural characteristics and stress redistribution. The net effect was enhancing the strength and flexibility of materials, the pure composition, controllable parameters, and simple procedures without altering the special properties of the substrate materials [167], [168]. Hydrophobic materials commonly exist on the surfaces of plants and anti-wetting animals, such as plant leaves covered with epicuticular wax or other substances, which are quite waterproof. A biomimetic super-hydrophobic surface has been fabricated by directly spraying a self-assembled nanomaterial inspired by hydrophobic properties found in nature [167,168]. A nano-silica gel with low surface energy was used to modify the multi-scale microstructure and surface chemical properties. Following the completion of the process, the roughness and self-breathing properties of the modified surface were significantly improved [167,168]. Based on the experimental data, the approach could be employed to modify various substrates. The water repellent is beneficial for simplicity in fabrication, high adaptation to a concrete-based substrate, cost-effectiveness, and self-breathability.

Innovative biopolymers from plant biomass and by-products from fishery waste have been developed for eco-friendly food-package films to reduce pollution and protect the environment by substituting the use of fossil fuel-derived compounds [167,168]. Marine organisms such as mussels can produce viscoelastic materials made from polymeric protein polysaccharides, which inspired research groups to produce novel materials [169]. Drawing from past successes, it is recommended that the future design and development of new materials be bio-inspired to enhance the ecological compatibility of farming structures, equipment, and systems. The proposal is justified considering bio-inspired materials have been developed to extract lanthanides and actinides and remove dyes and ingredients in nutraceuticals [170,171,172]. However, the transition from traditional to bio-inspired materials creates challenges and opportunities. Researchers have a role to play in maximizing the benefits and limiting the negative effects.

### 3.2. Biomimetic Structures 

Biomimetic innovations and materials have an integral role in the performance of agricultural structures such as greenhouses, storage facilities, and silos [173,174]. In contrast to conventional structures, agricultural structures must satisfy certain requirements such as adequate ventilation, the regulation of solar radiation, and thermal losses [175,176,177]. The design of greenhouse structures and ventilation is important to control the internal micro-climate, especially the temperature and the humidity, among other parameters, that predict crop growth and yield [155,178,179,180]. Bio-inspired methods of complex branching, metaheuristics, and streamlining have been described to optimize ventilation and sunlight in a greenhouse [181]. Anthill mounds have been examined to design a natural ventilation system for greenhouses and bricks [173]. Termite colonies are responsive to environmental changes and can survive as one system. As the research on termite mounds and geometry indicates, the natural ventilation is driven by pressure differences on the surface through the boundary layer of the wind. For this reason, digital simulation and experimentation have been applied to define and develop forms for natural branched ventilation in buildings [182]. The broad scope of the application demonstrates how biomimetic innovations, digital techniques, and complex branching morphology can be applied for natural ventilation and solar optimization in agricultural structures.

### 3.3. Resources Management—Solar Energy Harvesting

Agriculture relies heavily on energy; thus, the use of sustainable energy technologies in climate change is an urgent need. Renewable energy such as solar energy can reduce a farm’s electricity and heating costs [183]. The efficient use of photovoltaics (solar electric panels) can power farm operations, livestock buildings, greenhouses, and water pumps [184,185,186]. Intelligent solar tracking systems mimic the plants’ response to the sun’s movement, a process called phototropism, to maximize the energy collection from the sun. A solar tracking system is used to track the sun’s position across the sky to keep the photovoltaic system in the best position [187]. A novel solar tracking generation system was designed and tested to generate power for lighting in greenhouses. The experimental results indicated that the system could provide nearly 100% of the greenhouse power requirements [188,189,190,191]. However, the realization of optimal performance was contingent on optimizing the systems using IoT-based artificial neural networks to control solar tracking and precision agriculture systems [192,193]. An increase in the efficiency of solar systems can be achieved using hybrid techniques, neural network principles, and new artificial intelligence techniques [194]. A notable example is a solar tracking system based on computer vision combined with control algorithms developed in Mathematica and Simulink and the implementation of this method in a real system, based specifically on deep Convolutional Neural Networks (CNNs) for object localization and detection, inspired by biological processes where the connectivity pattern between neurons resembles the organization of the visual cortex [195]. Other phototropic systems can autonomously and instantaneously detect and track light in three-dimensional space at ambient temperatures very accurately and fast, without auxiliary supply or human intervention, such as the SunBOT (sunflower-like biomimetic omnidirectional tracker) [196,197]. This phototropic material mimics the asymmetric biological growth of plants and the elegant agility of living systems, which could provide a solution to energy harvesting and could lead to self-sustained, autonomously capable learning soft robots for performing tasks in several environments.

### 3.4. Water Resource Management

Commercial agriculture is key to the sustenance of modern civilizations, but resource use remains a challenge. At present, global food security is threatened by poor soil nutrition, unsustainable agricultural practices, and the excessive use of water in irrigation [16,180,198,199]. Conservative estimates suggest that agricultural activities account for 70% of global water use [200]. If the current rates are sustained, water scarcity will be experienced in most agriculturally productive regions due to climate change and global warming [42,201,202]. Considering excessive water use contributes to global water stress, agriculture institutions should collaborate across sectors to mitigate the challenge. The imbalance between the supply and demand for freshwater (water scarcity) and inadequate rainfall patterns (water shortages) already affects every continent and must be seriously considered. 

Runoff water can help complement alternative water sources for irrigation. A pilot project used surface runoff to offset the negative effects of a dry spell on cowpea farming in Nigeria’s savannah belt [203]. Similar techniques were employed in micro-catchment water harvesting for agriculture. A study conducted by researchers at the Freie Universität Berlin affirmed the suitability of surface runoff micro-catchment in reducing water stress in arid and semi-arid to sub-humid zones [204]. The capture and storage of surface runoff help to meet the water needs during the dry season or mitigate the impact of dry spells.

Novel methods for effective water harvesting have been developed based on nature-inspired designs, including the biomimicking spider webs to harvest fog water with electrospun polymer fibers [205]. The use of fog water for agriculture has not been widely explored except for the Warka tower project [206]. The spider web is nature’s example of how fibers can collect water droplets from fog. Mimicking this natural process to create nano- and micro-polymer webs with unique fiber structures opens new paths in fog-water collection. The Warka Tower is inspired by Namib bugs and lotus flower leaves, it is about 9 m tall, consisting of locally sourced materials such as bamboo, and its efficiency can be increased in the desert between sunset and sunrise, where the temperature range is huge [207]. However, the existing meshes used in collectors need improvement, as they show inadequacies in the surface of microstructure and property design [205]. Several species can directly harvest fog or dew, such as beetles, frogs, lizards, spiders, and plants. The design of the building of Namibia University Water Science Center was inspired by the Namib Desert darkling beetles (*Tenebrionidae*) [208]. The building is located behind a curved nylon wall that faces the ocean and catches the moisture from the ocean air. The wall’s net surfaces are shaped like the bumpy structure of the beetle’s shell, and the water is stored in underground water reservoirs. This design mimics the desert beetles, which can live in the harshest desert climate because they can harness water from the ambient air, using their bodies as “fog collectors assuming a characteristic fog-basking stance” [208]. Scanning electron microscopy studies of the beetles confirmed the presence of hydrophilic sites, which were instrumental in the collection of fog water. The analysis of the bio-inspired desert beetle water collection capabilities by Nørgaard and Dacke [208] was in agreement with Zhai et al. [209]. In the latter case, the researchers developed bio-inspired surfaces with super-hydrophilicity properties; this was achieved through the customized deposition of multilayer films on the hydrophilic patterns [209]. The films were made of poly(allylamine hydrochloride), poly(acrylic acid), and silica nanoparticle/semi-fluoro silane, which has a strong affinity for water molecules. Such milestones could help inform the development of new greenhouse-covering materials that harvest water using similar techniques as the Namib Desert darkling beetles. Various methods for preserving water have been developed, although those that are environmentally friendly and inexpensive should be applied. The main concern is that the bio-inspired design for fog and rainwater harvesting has not been employed on a wider scale. The documented pilot projects focused on communal benefits such as access to potable water rather than the exploitation of the bio-inspired designs for intelligent agricultural purposes [210,211]. However, there is immense potential for the Namib Desert darkling beetle’s bio-inspired designs for water harvesting for agricultural activities in water-scarce regions. 

## 4. Future Prospects

Despite concerns about costs and ecological impact, there are future prospects for utilizing robot bees/swarm robotics and soft robotics [56,119,121,123,124]. The positive outlook is reinforced by the current realities in the agricultural sector. The pollination of plants with natural bees has been compromised by anthropogenic pollution [121,123,124], especially the use of synthetic pesticides [122]. The intrinsic value of the robotic innovations is further reinforced by the projected market value of soft robotics. The sector recorded a 10% yearly growth, translating to $81 billion in market value [108]. The cumulative value of the industry would increase exponentially with innovations. For example, the current models of robot bees rely on a specially applied gel to facilitate crop pollination, and scientists have not coordinated large swarms of bees, despite concerted efforts to mimic bees. The autonomous or remote coordination of the bees would facilitate the large-scale employment of the robot bees. It would be easier to find pollen sites and pollinate the target plants while minimizing the risk of robot bees’ collisions [122]. The refinement of robot bees’ technology would translate to wide-scale adoption and better yields through robotic pollination. 

Beyond swarm and bee robotics, soft robotics has promising applications in fruit harvesting, considering the grip force could be adjusted to prevent the damage of fruits with a soft rind [84]. The refinement of the soft robotic technology would lead to the replacement of nearly all mechanical harvesters. The transition from mechanical harvesters and harvesting to soft robotic grippers would reduce harvest and post-harvest losses associated with the bacterial damage of mechanically damaged fruit, surface bruises, rupture, and crushing, the destruction of plant tissue, and plastic deformation. The process would also enable farmers to control the harvesting process precisely and achieve better yields. So far, FEA robotic grippers and FEA-tendon-driven grippers have been proven useful in harvesting tomatoes, cucumbers, bananas, apples, grapes, olives, and broccoli, among others [86]. On the downside, technology-related advances have not resolved concerns about information accessibility and implementation on a broader scale, the prospecting of suitable biomimicry patterns, and bio-inspired technologies’ relevance to the problem.

## 5. Conclusions

The growth of biomimetic innovations has been motivated by population growth, labor shortages on farms, and the desire to reduce the negative ecological impact on the environment. The projected surge in the global population is expected to introduce new challenges including food scarcity, water shortage, and higher emissions. Biomimetic solutions have been explored to enhance agricultural productivity through adaptation to nature. Sustainable agricultural production can meet the global food requirements through an efficient, inclusive, and resilient system. The integration of bio-inspired innovations enables large farms to achieve better efficiency and productivity while minimizing the adverse environmental impact; this was demonstrated with soft robotics for fruit and vegetable harvesting and swarm robotics. First, the soft robotic systems minimized the risk of mechanical damage, which is a common problem with traditional mechanical harvesters and hand-picking. The mitigation of mechanical damage (surface bruises, rupture, the crushing destruction of plant tissue, and plastic deformation) has practical benefits for farmers because post-harvest losses have a negative effect on profitability. Second, the swarm robotic systems could enhance pollination by complementing natural bees, which are critically endangered. Alternatively, the swarm robots could perform other important functions on farms including spraying pesticides and monitoring crop growth. Based on existing studies, it is clear that farmers have not yet exploited the full potential of soft and swarm robotics. The use of FEA robotic grippers and FEA-tendon-driven grippers in harvesting tomatoes, cucumbers, grapes, olives, and apples is inadequate.

The inability to exploit the full potential of the swarm and soft robotics could be partly attributed to a lack of consensus and phases of innovation. The soft and swarm robot systems developed by Arugga AI Farming, Bird Gard Australia, Boston Dynamics, and the Robotics Traction Unit (RTU) are not widely available. Various hypotheses were advanced to explain the phenomena including the cost of the technology, consumer attitudes toward the unknown, ecological concerns, and technical constraints. For example, Boston Dynamics had not created an effective robotic herding algorithm that can function optimally with a large herd of animals and nature and build robot-to-animal interactions. Other concerns relate to the practicality of swarm robotics. Critics noted that it is illogical to assume that humans could replicate the unique behavior of bees, which have been refined through evolution over 120 million years; this means it is not practical to replace natural bees with robots. However, other bio-inspired innovations have found broad acceptance including the water harvesting from fog, inspired by the Namib Desert darkling beetles (*Tenebrionidae*), and biomimetic materials, which exhibit unique relaxation, dynamic, multi-functional, and hierarchical properties for lightweight and durable applications.

Despite the concerns against soft and swarm robots, their role in agriculture cannot be disregarded considering the adverse effect of population growth and climate change on agriculture. The literature reviewed affirmed that biomimetic innovations are integral to climate-smart agriculture, which seeks to boost food security while minimizing carbon footprints. However, there is no consensus among researchers on achieving the goals. Conversely, pro-innovation researchers advocate for the development and deployment of robot bees to complement natural bees in agriculture. On the other hand, critics argue that the robot bees would negatively affect nature, given they are an invasive species. Additionally, the cost and practicality of robot bee projects remain questionable. 

Despite the challenges, the modernization of agriculture and the transition to agriculture 4.0 will continue, given it is necessary to increase economic growth and boost productivity. Presently, significant progress has been made in achieving interoperation among machine learning, robotics, and big data entities and using materials that are more sustainable and resilient. Based on conservative projections and the progress made by industry pioneers such as Arugga AI Farming, Bird Gard Australia, Boston Dynamics, and the Robotics Traction Unit (RTU), soft and swarm robotics will complement existing smart agricultural practices, leading to sustainable production. Nonetheless, the concerns raised by scholars about the life-cycle of man-made equipment in the natural environment cannot be disregarded and should be resolved through research and development. 

## Figures and Tables

**Table 1:** Soft gripper technology, gripper type, size, lifting ratio, scalability and controllability, response time, and surface conditions [86].

Soft Technology	Grasped Object	Object Size or Weight	Gripper Type	Gripper Size	Lifting Ratio	Controllability /Scalability	Response Time	Surface Condition
FEAs	Lettuce	250 × 250 mm	Two pneumatic actuators and a blade	8000 g, 450 × 450 × 300 mm	-	Close-loop with force sensor feedback/Yes	31.7 s	-
	Apple	-	Three soft finger design	Two fingers length: 95.25 mm One Finger length: 152.4 mm	-	Open-loop/-	7.3 s	-
	Mushroom	-	Three soft chambers in circular shell	Chamber height: 20 mm Chamber arc angle: 60^o^	30	-/Yes	-	Any surface
	Apple, Tomato, Carrot, Strawberry	69 mm, 5–150 g	Magnetorheological gripper	-	-	PID/-	0.46 s	Any surface
	Cupcake liners filled with peanuts	34–64 g	Three soft finger design	Finger size: 82 × 16 × 15 mm	-	FE Analysis/Yes	-	-
	Cupcake liners filled with red beans, higiki, ohitashi	75.2 g	Soft fingers	Finger length: 97 mm	1805%	Open-Loop/Yes	10 s pick and place (total procedure)	-
	Defrosted broccoli	33.54 × 23.94 mm, 3.8–7.0 g	Two soft fingers	Actuator size: 50 × 20 mm	-	-/-	3 s for inflation	-
	Granular kernel corn, Chopped green onion, Boiled hijiki	0.77–26.6 g	Four soft fingers	Finger size: 43 × 61.5 mm	-	Open-Loop/Yes	-	Any surface
	Orange	1000 g	Soft fingers	Finger size: 95 × 20 × 18 mm	-	Open-Loop/Yes	-	Any surface
	Tomato, Kiwifruit, Strawberry	45–76 mm	Four soft chambers in circular shell	Internal diameter: 46 mm Height: 30 mm	-	Open-Loop/Yes	2–5 s	Any surface
Tendon-driven	Tomato	500 g	Three soft finger design	-	-	Preprogrammed rotation of motors /Yes	-	-
	Tomato, Cucumber (slices) Avocado (Strips) Cherry Tomato, Olives, Pineapples cubes, Broccoli	-	Quad-Spatula design	-	-	-/Yes	-	Flat surfaces
FEA-Tendon-driven	Banana, Apple, Grapes	2700 g	Three soft finger design with a suction cup	389.69 g	7.06	Teleoperation Control/Yes	0.094 s (Rise time)	Any surface
Topology optimized soft actuators	Apple, Grapefruit, Guava, Orange, Kiwifruit	1499 g	Two compliant fingers	-	-	Open-loop (Arduino)/Yes	-	-

**Table 2:** Comparative analysis of fruit damage using mechanical harvesters, robotic grippers, and handpicking [84].

Cultivar	Harvest Method	Key Observations
Blueberry	Commercial mechanical harvester	Three out of four mechanically harvested blueberries were severely bruised and damaged by the commercial mechanical harvester.
	Handpicking	Nearly one in four hand-harvested blueberries had noticeable bruise damage.
Apple	Shake-and-catch harvesting system	At least eight percent of the three cultivars led to fruit bruises.
	Robotic picking using a three-finger gripper	If the robotic finger gripper’s grasping pressure and force are properly programmed, the risk of mechanical damage is reduced. Significant bruising of apples (46–60% of the harvest) was observed at higher grasping forces (14.5 to 15.9 N) 46.7% and grasping pressure (0.28 and 0.29 MPa). Based on the data, proper adjustment of the pressure and force is essential to minimize fruit damage.
	Handpicking	The risk of severe bruise damage on plants was mitigated if the average grasping force (5.05 N) and grasping pressure (0.24 MPa) were maintained at 5 N and 0.24 MPa. However, it is challenging for human hands to exert constant pressure and force during the entire harvesting process; bruise damage is unavoidable in handpicking fruits and vegetables.
Table olive	Manual picking	Manual picking by hand was responsible for 17.5–51% of the severe bruise damage.
	Trunk shaking harvester	There was a 62–77% % risk of damage if the farmers used mechanical trunk shakers.
	Grape straddle harvester	The risk of bruising damage was the highest, at between 91% and 100%.
Prune	Straddle mechanical harvester	Less than 10% of the prunes harvested using mechanical techniques showed signs of bruise damage.
	Handpicking	∼50% bruise damage
Plum	Straddle mechanical harvester	∼18% of the plums showed some bruise damage.

**Table 3:** The link between different swarm behaviors, application and adoption, and environment [116].

Environment	Project/Product Name	Basic Swarm Behaviors	Availability
Aerial	Distributed Flight Array	Self-assembly, coordinated motion	n.a.
	Crazyflie 2.1	Aggregation, collective exploration, coordinated motion, collective localization, collective perception	Open-source, commercial
	Finken-III		n.a.
Aquatic	CoCoRo	Aggregation, collective exploration, collective localization, task allocation	n.a
	Monsun		
	CORATAM		Open-source
Outer Space	Swarmers	Collective exploration, collective localization	n.a
	Marsbee	Collective exploration, coordinated motion, task allocation	

**Table 4:** Apples, cherries, and grapes losses linked to bird damage [140].

Crop	Yield per Acre	Annual Bird Management Costs	Current	Percent Lost to Bird Damage	
				No Management (Low Estimate)	No Management (High Estimate)
Wine Grapes	5.11	$1570	6%	36%	39%
Blueberries	5191	$404	12%	52%	54%
Tart Cherries	7260	$510	9%	43%	47%
Sweet Cherries	3.40	$692	31%	60%	67%
HC Apples	679	$249	5%	13%	15%

## Data Availability

Not applicable.
